# A Data-Driven Strategy for Identifying Individuals Resistant to SARS-CoV-2 Virus under In-Household Exposure

**DOI:** 10.3390/jpm12121975

**Published:** 2022-11-30

**Authors:** Roni Hen Gabzi, Tal Patalon, Noam Shomron, Sivan Gazit

**Affiliations:** 1 Faculty of Medicine, Tel Aviv University, Tel Aviv 69978, Israel; 2 Edmond J Safra Center for Bioinformatics, Tel Aviv University, Tel Aviv 69978, Israel; 3 Tel Aviv University Innovation Laboratory (TILabs), Tel Aviv University, Tel Aviv 69978, Israel; 4 Kahn Sagol Maccabi (KSM) Research & Innovation Center, Maccabi Healthcare Services, Tel Aviv 69978, Israel; 5 Maccabitech Institute for Research and Innovation, Maccabi Healthcare Services, Tel Aviv 69978, Israel

**Keywords:** COVID, SARS-CoV-2, household, electronic medical records, infectious diseases, resistance

## Abstract

This report describes the development of a data-driven approach for identifying individuals who tested negative to a SARS-CoV-2 infection, despite their residence with individuals who had confirmed infections. Household studies have demonstrated efficiency in evaluating exposure to SARS-CoV-2. Leveraging earlier studies based on the household unit, our analysis utilized close contacts in order to trace chains of infection and to subsequently categorize TEFLONs, an acronym for Timely Exposed to Family members Leaving One Not infected. We used over one million anonymized electronic medical records, retrieved from Maccabi Healthcare Services’ centralized computerized database from March 2020 to March 2022. The analysis yielded 252 TEFLONs, who were probably at very high risk of infection and yet, demonstrated clinical resistance. The exposure extent in each household positively correlated with household size, reflecting the in-house rolling transmission event. Our approach can be easily implemented in other clinical fields and should spur further research of clinical resistance to various infections.

## 1. Introduction

Since the SARS-CoV-2 pandemic outbreak, susceptibility to the virus has been widely investigated from both genetic and clinical aspects [1,2,3,4]. Genome-wide association studies have identified an association between the ABO locus and innate resistance to infection, though the protective effect is small [5,6,7]. In vitro studies have pointed to several other candidate genes that might provide protection, such as a rare variant in the ACE2 receptor [8]. Other multi-OMICs (such as proteomics and metabolomics studies) have yet to be published on this subject.

A different approach to susceptibility explores the sub-clinical infection hypothesis. These studies examined whether in some individuals, exposure leads to a subclinical infection without seroconversion, that is, without a positive reverse transcription polymerase chain reaction (RT-PCR) or antibody measurement; and whether this results in clinical resistance, rather than a ‘complete’, or measurable, infection [1,5]. Based on this hypothesis, we aimed to develop a feasible and modifiable approach for identifying TEFLONs, an acronym for Timely Exposed to Family members Leaving One Not infected. TEFLONs’ high probability of clinical resistance to a SARS-CoV-2 infection is based on their not contracting SARS-CoV-2 infection, despite residence in a household in which at least one resident had a confirmed infection. We used the household unit for the measure of exposure as it has demonstrated efficiency in assessing close contact to infected persons, and has confirmed exposure to SARS-CoV-2 infections with a high degree of certainty [9,10].

We expect our approach to be suitable for global implementation, regardless of the accessibility to genetic or other OMIC testing. This study describes the application of the approach to identifying TEFLONs, in a database of one million anonymized electronic medical records (EMR). We searched for possible commonalities and similar patient journeys of the TEFLONs, which could elucidate their potential non-genetic resistance to SARS-CoV-2.

## 2. Methods

### 2.1. Nomenclature and Definitions

Individuals without a previous record of a positive RT-PCR or antigen test for SARS-CoV-2, or a positive serology for SARS-CoV-2, were referred to as “SARS-CoV-2 naïve”. Contrastingly, those with a previous documented SARS-CoV-2 infection, determined by a positive RT-PCR test, were referred to as “previously infected”, “convalescent” or “recovered” individuals. Persons who received at least two doses of the BNT162b2 vaccine were referred to as “vaccinated individuals”. Those who were both previously infected and vaccinated were referred to as having “hybrid immunity”. A person exposed but not infected, the target population of our research, was referred to as a TEFLON.

**Figure 1:**
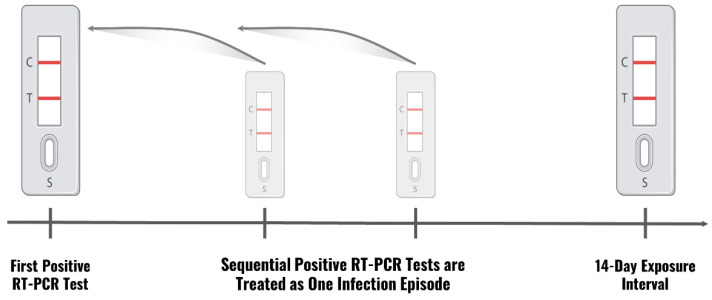
Representation of an Individual Positive Event. The pivotal event was considered the first positive reverse transcription polymerase chain reaction (RT-PCR) test for an individual (left test illustration), and each positive test in the following 14-day period (light test illustrations) was treated as the same infection event.

**Figure 2:**
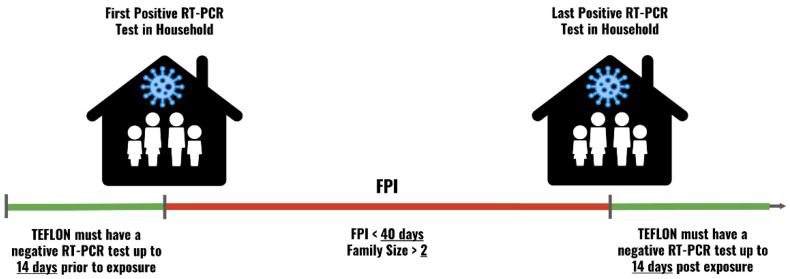
The minimal set of criteria that defined TEFLONs in relation to exposure events in the household, or the Familial Positive Interval (FPI). The horizontal line is comprised of three time frames. The first period (green) was denoted by the pre-exposure negative reverse transcription polymerase chain reaction (RT-PCR) test date, which had to fall within a 14-day interval prior to the exposure event, or the FPI. The middle period (red) was the duration of the FPI. The final period was defined by the time between the last documented exposure to a household member, and a TEFLON’s negative RT-PCR test, or proof of non-infection (green).

An Individual Positive Event (IPE) describes a 14-day interval during which sequential positive RT-PCR tests of the same person are treated as a single infection episode. The starting time of such is the date of the first positive RT-PCR test in the series. For example, if an individual had three positive RT-PCR tests over the course of 10 days, the earliest positive RT-PCR test would be defined as the starting time of the IPE (Figure 1). A Familial Positive Interval (FPI) denotes the time between the first and last positive RT-PCR tests in each household (Figure 2).

### 2.2. Data Sources

This study used anonymized EMR, retrieved from the centralized computerized database of Maccabi Healthcare Services (MHS), for the period of March 2020 to March 2022. Israeli citizens are required to choose one of four state-mandated, non-for-profit national health funds, for health care provision. Health funds are prohibited by law from denying membership to any resident. MHS is the second largest health fund. The 2.6 million members comprise 26% of the national population, and provide a representative sample. MHS has maintained a centralized database of EMRs for over thirty years, with less than 1% disengagement rate among its members. This enables comprehensive longitudinal medical follow-up. The centralized dataset includes extensive demographic data, clinical measurements, outpatient and hospital diagnoses and procedures, medications prescribed, imaging performed, and laboratory data that are analyzed in a single central facility.

### 2.3. Data Extraction

Individual-level data included the year of birth, biological sex, family connections and a coded residential socioeconomic geographical statistical area. The latter is assigned by Israel’s Central Bureau of Statistics; the smallest geostatistical unit of the Israeli census (corresponds roughly to neighborhoods) and embodies socioeconomic status. Extracted data also included medical history, namely previous diagnoses and procedures, and chronic diseases from MHS’s automated registries, including cardiovascular diseases [11], hypertension [12], diabetes [13], chronic kidney disease [14], chronic obstructive pulmonary disease, obesity (defined as a body mass index of 30 kg/m^2^ or higher) and immunocompromised conditions. COVID-19 related information consisted of the dates of all RT-PCR tests for SARS-CoV-2 (which are recorded centrally), and vaccination dates and COVID-19 related hospitalizations and mortality.

### 2.4. Measured Outcome

We had two overarching goals. The first goal was to develop criteria for defining TEFLONs, which would be efficient enough for global implementation, regardless of accessibility to genetic or other OMIC testing. Our second goal was to explore the possibility of discovering in the EMR, commonalities of the TEFLONs that might shed light on the source of their potential non-genetic resistance to SARS-CoV-2.

### 2.5. Study Design—Ensuring High Probability of Exposure

The first step in identifying TEFLONs (persons presumably exposed-but-uninfected) was to ensure a high probability of exposure. Household studies that have been conducted to assess SARS-CoV-2 infectiousness and vaccine effectiveness have presumed exposure according to residence with an infected person [9,10]. We adopted the household unit because of its efficiency. Moreover, confirming exposure from an outside source is challenging, even when the region is restricted to a small residential radius.

### 2.6. Study Design—Defining TEFLONs, the Target Population

To be designated as a TEFLON, an individual was required to be SARS-CoV-2 naïve, meaning without a record of a positive RT-PCR, antigen or serology test, throughout the study period. Next, to ensure high probability of exposure, the individual had to be a member of a household that included at least one infected individual. After exposure was confirmed, we ensured that the potential target population was indeed uninfected, rather than under-detected (not tested) around the time of the infectious exposure. Outlining a timeframe for an infectious period within a household is challenging, as was demonstrated in our research on devising a binomial model for household transmission [10]. Specifically, a temporal window must be defined, during which it is reasonable to assume that a household member was infectious. If too short, this interval will be biased to exclude members; if too long, it will falsify an association between a negative test and a confirmed exposure. Therefore, we obliged each potential TEFLON to have at least one negative RT-PCR test up to 14 days prior to the exposure-event and a negative RT-PCR test up to 14 days subsequent to the exposure-event. The 14 days prior the event were designated due to the possibility of a latent and prodromic period of the infected member, which likely occurred before people were prompt about testing (Figure 2).

In addition to the above, we had to account for households of more than two members, with a possible chain of transmission and infections. An example of such is a household in which two members are infected sequentially, and a third member remains uninfected. In such instance, the FPI (a multi-member ‘exposure event’) is defined as the interval between the first infected and the last infected person in the household. However, the negative RT-PCR is still mandated 14 days pre-FPI and 14 days post-FPI, for the potential TEFLON member of the household. Moreover, to increase the likelihood of referring to the same rolling event, or chain of transmission, we limited the FPI exposure event to a maximum length of 40 days from the first to the last positive RT-PCR in the household. This criterion was intended to ensure that a positive event does not actually represent two or more separate events in which a TEFLON had only one confirmation of a negative RT-PCR, regardless of a pre-event or post-event confirmation of resistance. An example of such in a candidate TEFLON, would be a positive event that was preceded by a negative test, and that was followed by another positive event one year later, 14 days after another negative test. In developing criteria for TEFLONs, we also attempted to establish a minimum FPI of 14 days. This was intended to distinguish a rolling event from a single event. However, this criterion did not alter the number of identified TEFLONs.

### 2.7. TEFLONs and Vaccination Status

We examined the vaccination statuses of the TEFLONs and their household members. The rationale was that being unvaccinated, partially vaccinated or in the waning phase of the vaccine protection could support or confound a possible innate trait of resistance [15]. Immune status was determined on the first day of exposure. Similarly, we examined the household’s vaccination status, as breakthrough infections (infections in fully vaccinated individuals) were previously demonstrated as containing lower viral loads [16], and thus possibly less infectious. If the latter is true, and most household members (and potential transmitting agents) were fully vaccinated at the time of exposure, it could be argued that the TEFLON’s resistance challenge was somewhat moderated.

### 2.8. Searching for TEFLON Commonalities

After defining TEFLONs, our aim was to seek common features within the EMR, of individuals fulfilling the criteria. Therefore, we searched for possible shared characteristics of TEFLONs, whether around the time of exposure (such as medications taken that might have increased resistance), or pertaining to life-long medical journeys (such as chronic diseases) or to baseline characteristics (such as age).

Following from the above, encompassing individual-level clinical data were extracted (see: Data extraction) and characteristics that could account for the exposure were compared. First, we examined demographic characteristics, namely age, biological sex, and familial structure (i.e., family size and the number that were exposed or had positive RT-PCR). Next, we explored documented diagnoses, acute and chronic ones separately. A chronic diagnosis was included in the analysis if it was recorded at least three months before the TEFLON’s first exposure to the IPE in the household. We included chronic conditions both from coded diagnoses and from registries that encompass a set of related conditions (such as immunosuppression conditions), extracted from MHS’s automated registry database. For acute diagnoses, we examined any infectious disease, recorded up to two months prior to the TEFLON’s exposure.

Medication usage data were also analyzed, separately by chronic and acute usage. Chronically prescribed medications were included in the analysis if they were first purchased prior to a TEFLON’s first exposure to a SARS-CoV-2 transmitting agent in the household. Medication for acute and transient conditions were included if they had been prescribed up to one month prior to the TEFLON’s presumed exposure.

## 3. Results

### 3.1. Ensuring High Probability of Exposure

**Figure 3:**
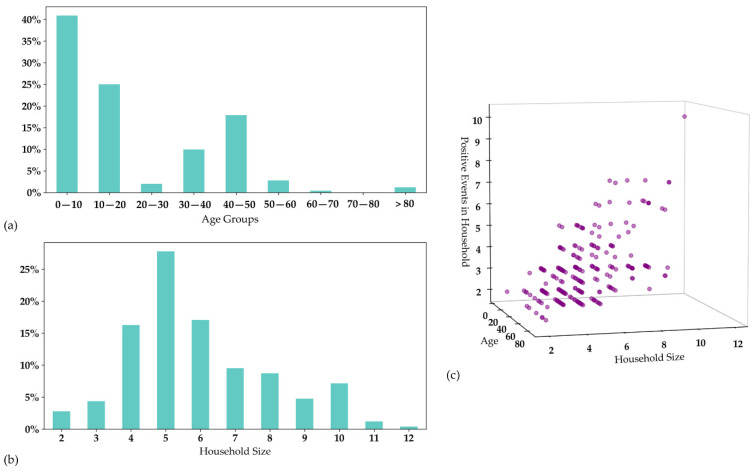
(**a**) The age groups of the people designated as TEFLONs (Timely Exposed to Family members Leaving One Not infected). The X axis shows the age groups. The Y axis demonstrates the relative proportion of each age group of the entire TEFLON population. (**b**) The household sizes of the TEFLONs. (**c**) The relation between household size and the number of positive events. The X axis shows household sizes, the Y axis shows the ages of the persons designated as TEFLONs and the Z axis shows counts of individual positive events in each household.

We first addressed household size as a measure of exposure, whereby most households were comprised of 2–3 members (Figure 3). Next, we calculated the number of IPEs in each TEFLON’s household, in relation to the size of the household. As anticipated, the number of positive events correlated positively with household size, thus reflecting the familial rolling event of transmission (Figure 3).

### 3.2. Defining TEFLONs, the Target Population

**Figure 4:**
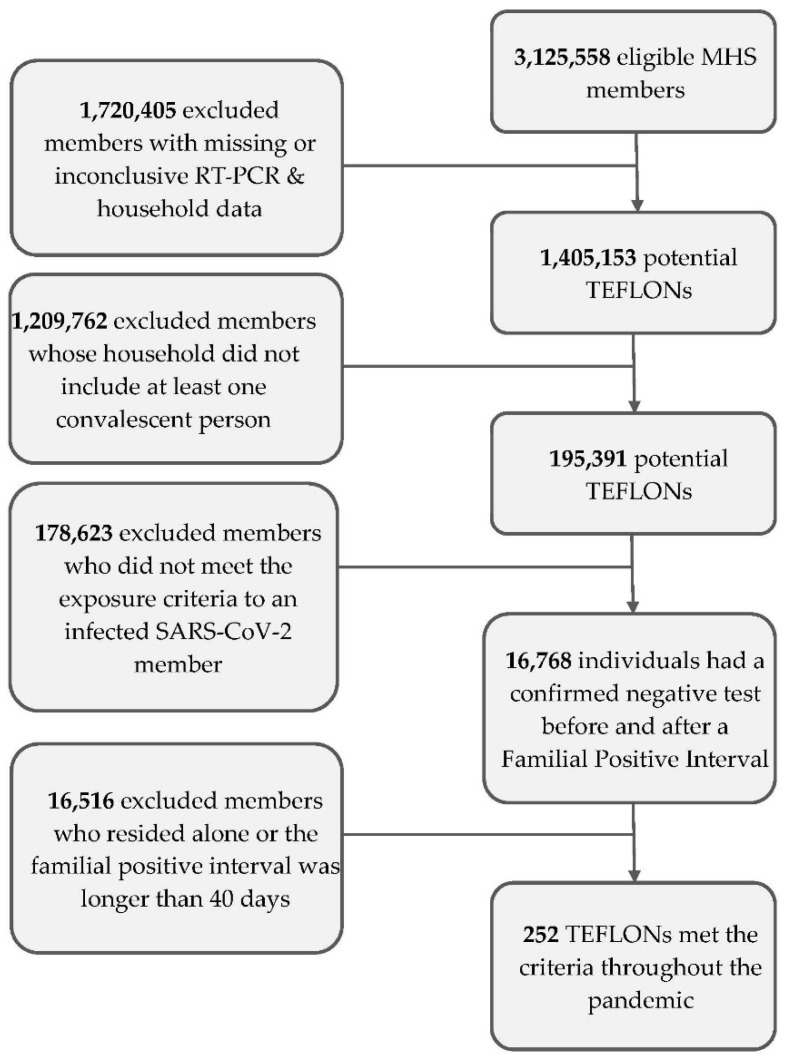
The data investigation process—The right side of the flow chart describes the included population at each step. The left side shows the criteria that were used at each step. MHS, Maccabi Healthcare Services; RT-PCR, reverse transcription polymerase chain reaction; TEFLONs, Timely Exposed to Family members Leaving One Not infected.

The data investigation started with all MHS members. First, 1,017,326 individuals were omitted from the analysis as they had missing or inconclusive data regarding household membership. Next, we excluded 703,079 members who had no record of RT-PCR testing, whether performed at MHS or through an external facility, as this precluded establishing their infection history. This initial screening yielded 1,405,153 potential TEFLONs. Then, we removed persons who were residing alone (precluding ensuring close contact or household exposure). We also omitted persons who lived in households that did not contain at least one convalescent person, as necessary to meet the exposed but uninfected criteria. This resulted in 195,391 potential TEFLON candidates. Of these, 16,768 had confirmed negative tests before and after FPI (household ‘positive interval’). Of those remaining, 252 had confirmed negative tests up to 14 days pre- and post-FPI (Figure 4).

**Figure 5:**
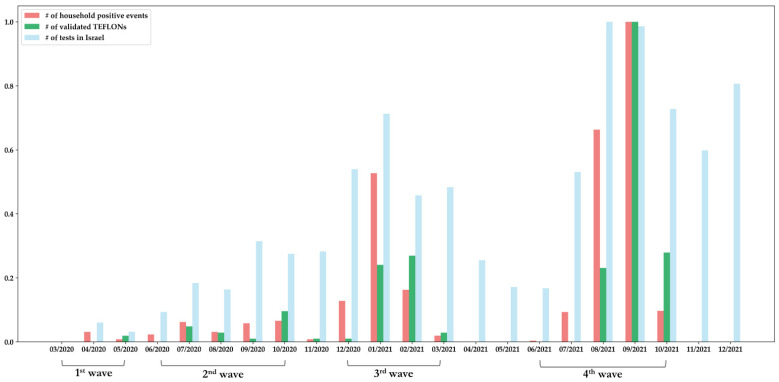
The timeline of SARS-CoV-2 waves in Israel, depicted by quantities of monthly tests [19] (light blue) compared to individual positive events (red) and the number of TEFLON (Timely Exposed to Family members Leaving One Not infected) validations per month. All the counts were scaled to a common 0–1 range.

These 252 individuals met the TEFLON set of criteria throughout the pandemic, from March 2020 to November 2021 (Figure 5). This period corresponded to the wild type, Alpha/Beta and Delta waves in Israel [17]. The Omicron variant was shown to escape the immune system more effectively than the other variants, both in studies of vaccinated individuals and of previously infected ones. Thus, we examined whether any of these 252 persons had records of positive COVID tests during the Omicron surge, which rapidly spread in Israel starting in December 2021. Forty-seven of the 252 TEFLONs had documentations of infections with SARS-CoV-2 during the Omicron surge, until March 2022. These documentations were only of rapid antigen tests, and not of positive RT-PCR tests, probably due to the changed health policy in Israel. Accordingly, as of 19 January 2022, RT-PCR tests were not readily available to persons under age 60 years [18], and instructions concerning who should undergo an antigen test performed by certified personnel, rather than at-home testing, changed repeatedly.

### 3.3. TEFLONs, Vaccination Status and Exposure

Of the 252 identified TEFLONs, 182 (72.2%) did not receive any vaccination shots before their first exposure to an infected SARS-CoV-2 household member, the date that defines the beginning of the FPI. Of these 182, 168 (92.3%) remained unvaccinated over the entire course of the FPI.

### 3.4. Searching for TEFLON Commonalities

The baseline characteristics of the 252 TEFLONs are summarized in Table 1, compared to their household members who had a confirmed SARS-CoV-2 infection during the study period. Over 85% of the identified TEFLONs were young; 166 (65.9%) were under age 18 years (Figure 3). No significant gender prevalence was found; 49.2% were females.

Comorbidities were uncommon in the TEFLON group, consistent with their young age. Of 252 individuals, only 1 had a cardiovascular disease, 3 were diagnosed with diabetes, none with COPD, 8 with CKD, 6 with hypertension, 2 with an immunosuppressive condition and 1 with inflammatory bowel disease (Table 1). No medication group was found to be particularly prevalent in the TEFLON group.

## 4. Discussion

While virus susceptibility has been widely investigated, our goal was to characterize individuals who show clinical resistance to infection under confirmed and sustained exposure. Here, we described the development of an approach for identifying individuals with a high probability of such clinical resistance. We used anonymized EMR to examine the history of SARS-CoV-2 infections within household units. This leverages earlier studies that demonstrated the household unit as an efficient measure, both for tracing in-house chain of infection and as a proxy for sustained and close contact. Indeed, the number of positive events in a household unit correlated with household size, thus reflecting the familial rolling event of transmission. Notably, we integrated RT-PCR, rapid antigen tests, and serology tests with the household affiliations and medical histories of over one million members of a national health fund.

Interestingly, the majority of TEFLONs did not receive any vaccination shots before their first exposure and most of them remained unvaccinated over the entire FPI. This result implies that vaccinations were not an influential component in TEFLONs’ resistance to infection. In addition, comorbidities were uncommon among TEFLONs, a finding that is probably related to the relatively young age of the TEFLONs.

Our study has some limitations worth mentioning. First, TEFLONs were characterized during the dominant periods of the wild type, Alpha/Beta and Delta strains of SARS-CoV-2 [17]. Later, 47 of the 252 TEFLONs were found to be infected with SARS-CoV-2 during the Omicron surge. The health policy applied during that period provided RT-PCR tests for the older population only, and thus the usage of undocumented self-testing kits increased. Altogether, the true Omicron-TEFLONs could not be inferred. A second limitation is that important parameters for assessing close contact are inherently missing from our data, such as the dimensions and numbers of rooms of the residential units, and the degree of meticulous quarantine enforced in each household.

High testing frequency, combined with reliable household information, were important components in gaining a high confidence level and mitigating our limitations to some extent. Certainly, these parameterized conditions can be adjusted to yield larger TEFLON populations if lower assurance is sufficient.

Investigating EMR data of clinically resistant populations can expand our knowledge of shared commonalities within those populations, provide better screening methods at pandemic times, and hopefully spur clinical efforts to characterize sub-groups at particular risks of infection.

## 5. Conclusions

The approach we developed can be easily implemented to characterize populations with clinical resistance to a SARS-CoV-2 infection in other global databases, and in other clinical fields. Implementation requires availability of close-contact measurement, such as the household unit; and of a lab test or diagnosis code, such as our use of the RT-PCR, antigen and serology tests, for the investigated disease-causing agent. In designating TEFLONs, we used stringent parameters to confirm exposure, and the presence or absence of infection. Accordingly, we included individuals who were all likely to be at very high risk of SARS-CoV-2 infection and yet seemingly had clinical resistance.

The observation that most TEFLONs were unvaccinated during the presumed exposure supports the possibility of an innate trait of resistance, rather than vaccination-derived immunity to the virus. Examination of neither medications nor disease history yielded shared features that could facilitate identifying TEFLONs.

## Figures and Tables

**Table 1:** Characteristics and exposing agents of the individuals included, by group: TEFLONs (Timely Exposed to Family members Leaving One Not infected) and non-TEFLON household members.

	Category	Non-TEFLON Household Members	TEFLONs	Missing	Overall
*n*		268,777	252		269,029
Age group, years, *n* (%)	0–18	133,972 (49.8)	166 (65.9)	0	134,138 (49.9)
	18–45	68,014 (25.3)	51 (20.2)		68,065 (25.3)
	45–60	45,330 (16.9)	31 (12.3)		45,361 (16.9)
	60–75	17,566 (6.5)	1 (0.4)		17,567 (6.5)
	>75	3895 (1.4)	3 (1.2)		3898 (1.4)
Biological sex, *n* (%)	Female	144,317 (53.7)	124 (49.2)	0	144,441 (53.7)
	Male	124,460 (46.3)	128 (50.8)		124,588 (46.3)
BMI, *n* (%)	Underweight (<18)	109,364 (40.7)	132 (52.4)	0	109,496 (40.7)
	Normal weight (18–25)	71,866 (26.7)	59 (23.4)		71,925 (26.7)
	Overweight (25–30)	50,085 (18.6)	38 (15.1)		50,123 (18.6)
	Obesity (≥30)	37,462 (13.9)	23 (9.1)		37,485 (13.9)
SES, *n* (%)	Low (1–3)	55,879 (20.8)	46 (18.3)	1	55,925 (20.8)
	Medium (4–6)	125,877 (46.8)	107 (42.5)		125,984 (46.8)
	High (7–10)	87,020 (32.4)	99 (39.3)		87,119 (32.4)
Vaccination status (# of shots), *n* (%)	0	219,933 (81.8)	182 (72.2)	0	220,115 (81.8)
	1	11,190 (4.2)	7 (2.8)		11,197 (4.2)
	2	30,411 (11.3)	56 (22.2)		30,467 (11.3)
	3	6970 (2.6)	7 (2.8)		6977 (2.6)
	4	273 (0.1)			273 (0.1)
CVD, *n* (%)		1645 (0.6)	1 (0.4)		1646 (0.6)
DM, *n* (%)		12,104 (4.5)	3 (1.2)		12,107 (4.5)
Hypertension, *n* (%)		23,206 (8.6)	6 (2.4)		23,212 (8.6)
CKD, *n* (%)		11,114 (4.1)	8 (3.2)		11,122 (4.1)
COPD, *n* (%)		1534 (0.6)			1534 (0.6)
IBD, *n* (%)		1490 (0.6)	1 (0.4)		1491 (0.6)
Immunosuppression, *n* (%)		3170 (1.2)	2 (0.8)		3172 (1.2)

BMI—Body Mass Index; SES—socioeconomic status on a scale from 1 (lowest) to 10; CVD—cardiovascular diseases; DM—diabetes mellitus; CKD—chronic kidney disease; COPD—chronic obstructive pulmonary disease; IBD—inflammatory bowel disease; vaccination status—up to the first exposure for TEFLONs and up to the first positive RT-PCR for previously infected individuals.

## Data Availability

According to the Israeli Ministry of Health regulations, individual-level data cannot be shared openly. Specific requests for remote access to de-identified community-level data should be directed to KSM, Maccabi Healthcare Services Research and Innovation Center. During analysis, we also used data retrieved from the Israeli Ministry of Health website [19].
